# Engaging Underrepresented Caregiving Communities in Dementia Research

**DOI:** 10.1093/geroni/igaa057.2773

**Published:** 2020-12-16

**Authors:** Andrea Gilmore-Bykovskyi, Ishan Williams

## Abstract

Family and friend caregivers of persons with dementia from underrepresented and traditionally underserved backgrounds are significantly underrepresented in dementia and caregiving research despite heightened disease risk, poorer outcomes, and disproportionate use of services within these populations. Efforts to develop and disseminate methods that foster greater inclusion of underrepresented caregiving populations in research are essential to ensuring that culturally specific understandings, priorities, and needs of these groups are systematically understood and addressed. In this symposium, we present a variety of studies that illustrate successful efforts to include dementia caregivers from underrepresented backgrounds in research. Two presentations focus on African American caregivers, one on caregivers residing in highly under-resourced areas, one on Latino caregivers, and one on sexual and gender minority (SGM) caregivers. The first presentation describes a capacity building approach through African American faith communities to develop a research registry and address informational needs regarding dementia. The second presentation focuses on eliciting African American caregivers experiences of crisis events. Presentation three describes a coalitional, community-informed approach to engaging caregivers in highly under-resourced areas to investigate experiences with post-acute care. The fourth presentation describes a community-network approach to implementing a text-message based support intervention among Latino caregivers; and the fifth presentation illustrates the utility of digital methods for engaging SGM dementia caregivers. Collectively, these presentations demonstrate a variety of approaches to engaging dementia caregivers from underrepresented and traditionally underserved backgrounds in research that are specific to individual communities and local contexts – as well as the findings that result from these efforts.

